# Supplementary material for: A Novel Deoxyribonuclease Low-Molecular-Weight Bacteriocin, Carocin S4, from Pectobacterium carotovorum subsp. carotovorum
Source: Microorganisms. 2023 Jul 22;11(7):1854. doi: 10.3390/microorganisms11071854 (PMC10386115; doi:10.3390/microorganisms11071854)
Supplement: Supplementary file 1 [file microorganisms-11-01854-s001.zip › Supplementary Figure S1.pdf]

Supplementary Figure S1

Indicator	T04	TO6	MR6	2J-1	86-H-2	86-M-1	89-H-4	J4	Ea1068	E108	CA7	H4	95F3	TT4	2T-2	07CA6	A100	CA2	R1	TN13	TN3	87-A-1	L3	RI7
TO4							●	●																
TO6			●				●	●			●		●		●	●	●	●	●	●	●	●	●	●
MR6		●			●	●		●	●		●		●	●		●	●	●			●	●		●
2J-1		●	●		●	●			●			●	●				●	●	●	●	●	●	●	●
86-H-2			●					●	●				●				●							●
86-M-1			●						●				●				●							●
89-H-4		●			●	●			●				●				●				●	●		●
J4		●	●			●						●	●			●	●	●	●	●	●	●	●	●
Ea1068																								
E108																								
CA7																								
H4																								
95F3																								
TT4																								
2T-2					●											●	●	●						●
07CA6																								
A100																								
CA2																								
R1																	●							
TN13																								
TN3									●								●							
87-A-1																								
L3		●			●	●			●				●				●	●			●			●
RI7																								
T-29																								

Cross-comparison of the bacteriocin activity of each strain against other indicator strains. Each dot (●) indicates that the bacteriocin produced by the strain has observable inhibition on the production of the bacteriocin of the indicator strain.
