# Supplementary material for: A Novel Deoxyribonuclease Low-Molecular-Weight Bacteriocin, Carocin S4, from Pectobacterium carotovorum subsp. carotovorum
Source: Microorganisms. 2023 Jul 22;11(7):1854. doi: 10.3390/microorganisms11071854 (PMC10386115; doi:10.3390/microorganisms11071854)
Supplement: Supplementary file 1 [file microorganisms-11-01854-s001.zip › Supplementary Figure S2.pdf]

Supplementary Figure S2

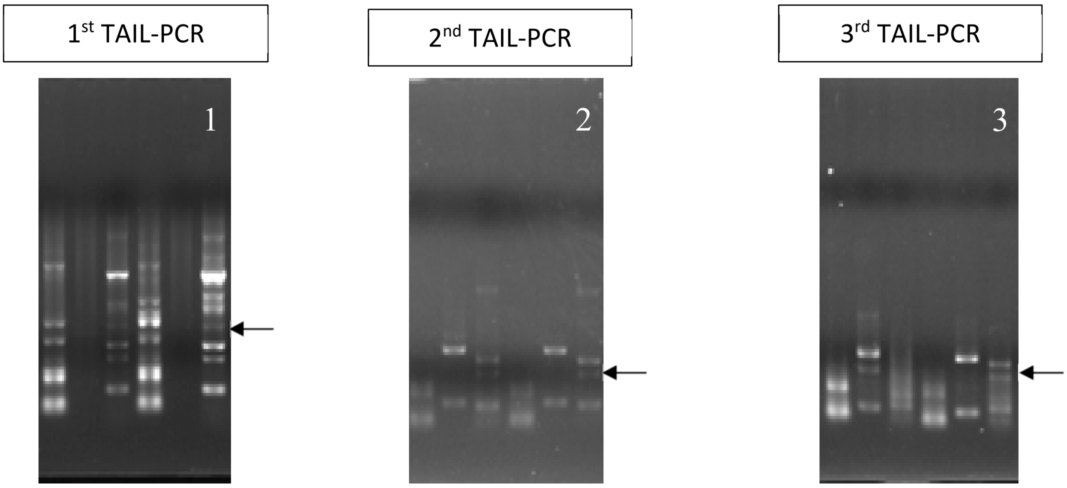

Using TAIL-PCR, the unidentified sequence fragments of the specific primer and the random primer are amplified. The DNA of the primary product became shorter following three TAIL-PCRs. (1) 1st TAIL-PCR; (2) 2nd TAIL-PCR; and (3) 3rd TAIL-PCR.
