# Supplementary material for: A Novel Deoxyribonuclease Low-Molecular-Weight Bacteriocin, Carocin S4, from Pectobacterium carotovorum subsp. carotovorum
Source: Microorganisms. 2023 Jul 22;11(7):1854. doi: 10.3390/microorganisms11071854 (PMC10386115; doi:10.3390/microorganisms11071854)
Supplement: Supplementary file 1 [file microorganisms-11-01854-s001.zip › Supplementary Figure S3.pdf]

Supplementary Figure S3

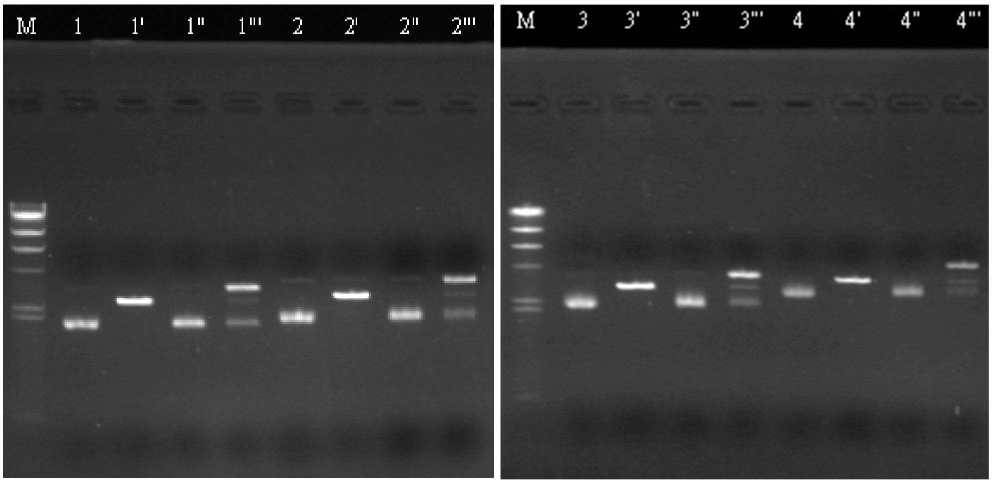

Supplementary Figure S3. Mass DNA (pMCL200, pGEM-4Z, pBluescript and pQE30) reacts with CaroS4K. (28℃/60 min)

M: λDNA /*Hind* III.

1 : pMCL200 (100 ng).

1’ : pMCL200 / *Sac I*.

1” : pMCL200 (100 ng) in 10 mM Ca^{2+} buffer without CaroS4K (1μM) .

1’” : pMCL200 (100 ng) in 10 mM Ca^{2+} buffer with CaroS4K (1μM).

2 : pGEM-4Z (100 ng).

2’ : pGEM-4Z / *Sac I*.

2” : pGEM-4Z (100 ng) in 10 mM Ca^{2+} buffer without CaroS4K (1μM).

2’” : pGEM-4Z (100 ng) in 10 mM Ca^{2+} buffer with CaroS4K (1μM).

3 : pBluescript (100 ng).

3’ : pBluescript / *Bsa AI*.

3” : pBluescript (100 ng) in 10 mM Ca^{2+} buffer without CaroS4K (1μM).

3’” : pBluescript (100 ng) in 10 mM Ca^{2+} buffer with CaroS4K (1μM).

4 : pQE30 (100 ng).

4’ : pQE30 / *Sac I*.

4” : pQE30 (100 ng) in 10 mM Ca^{2+} buffer without CaroS4K (1μM).

4’” : pQE30 (100 ng) in 10 mM Ca^{2+} buffer with CaroS4K (1μM).
