# Supplementary material for: A Novel Deoxyribonuclease Low-Molecular-Weight Bacteriocin, Carocin S4, from Pectobacterium carotovorum subsp. carotovorum
Source: Microorganisms. 2023 Jul 22;11(7):1854. doi: 10.3390/microorganisms11071854 (PMC10386115; doi:10.3390/microorganisms11071854)
Supplement: Supplementary file 1 [file microorganisms-11-01854-s001.zip › Supplementary Figure S4.pdf]

Supplementary Figure S4

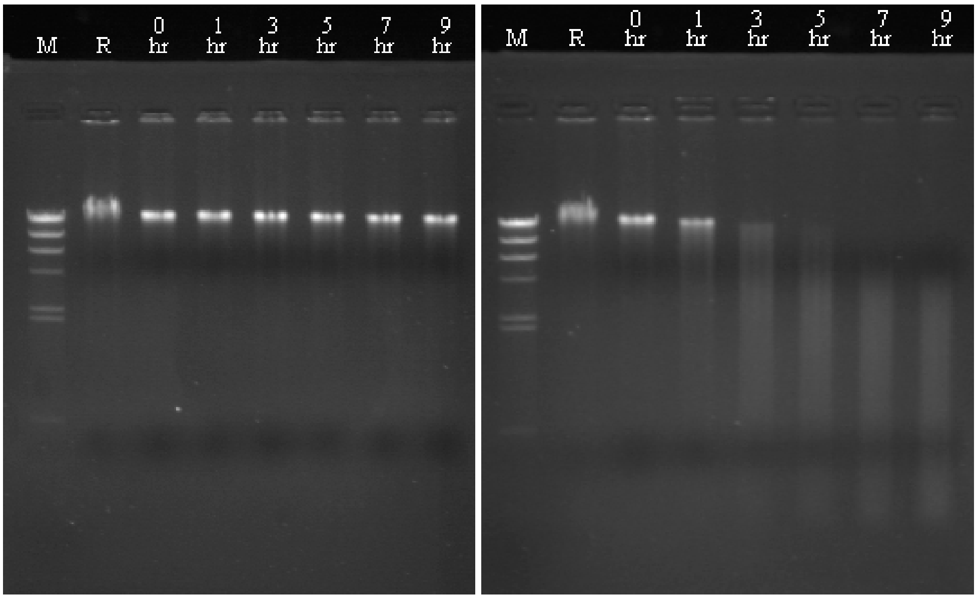

Without CaroS4K With CaroS4K

Supplementary Figure S4. Genomic DNA of 95F3 reacts with CaroS4K for different lengths of time.

The 1 mM Ca^{2+} buffer containing genomic DNA of 95F3 (100 ng) was incubated with or without CaroS4K (1 μM) for different time at 28℃. The reaction time was increased from 0 to 9 hours as indicated on the top of each lane.

M : λDNA /*Hind* III. R : genomic DNA of 95F3 (100 ng).
