# Supplementary material for: A Novel Deoxyribonuclease Low-Molecular-Weight Bacteriocin, Carocin S4, from Pectobacterium carotovorum subsp. carotovorum
Source: Microorganisms. 2023 Jul 22;11(7):1854. doi: 10.3390/microorganisms11071854 (PMC10386115; doi:10.3390/microorganisms11071854)
Supplement: Supplementary file 1 [file microorganisms-11-01854-s001.zip › Supplementary Figure S5.pdf]

Supplementary Figure S5

```
                   *        20         *        40         *        60         *
CaroS4K : MIKYRLYAPNDGDTMTVDGGGGGWVSNDDRKGGNDRNNDKGGSAVDYGKNPEKQTVINPYLFFAIPPAVYLI :  72
CaroS1K : ------------------------------------------------------------------------ :   -

                80         *       100         *       120         *       140
CaroS4K : DDVWGFTFNTAAIEVGLKKVADFALKATPVAGRLVGILGAIMPSDIAPDSIDPVFNGMQQTKMAQMAAQLAA : 144
CaroS1K : ------------------------------------------------------------------------ :   -

               *       160         *       180         *       200         *
CaroS4K : QKGVPNIGYSVTAMPADLVSSLPVSEISSLPTAPASLLAQSVINTELSRRQLTLTKPAVNAPVANIPVVKAE : 216
CaroS1K : ------------------------------------------------------------------------ :   -

           220         *       240         *       260         *       280
CaroS4K : KTAVPGVYSVKVIADKPALQIKLDKTQPALAKNPPKVKDDVQVSSFLSSPVADTHHAFIDFGSDHEPVYVSL : 288
CaroS1K : ------------------------------------------------------------------------ :   -

           *       300         *       320         *       340         *       360
CaroS4K : SKIVTAEEEKKQVEEAKRREQEWLLRHPITAAERKLTEIHQVLSFAQQLKESSTATISEKTKTVAVYQEQVN : 360
CaroS1K : ------------------------------------------------------------------------ :   -

                   *       380         *       400         *       420         *
CaroS4K : TAAKNRDNFYNQNRGLLSAGITGGPGYPIYLALWQTMNNFHQAYFRANNALEQESHLLNQARSDLSKAEQLL : 432
CaroS1K : ------------------------------------------------------------------------ :   -

               440         *       460         *       480         *       500
CaroS4K : AENNLLQVETERMLAEEKEIKRNRVNVSTFGTVQTQLSTLLSAFYAATSGSTASISQSVPSGELASFLYKPQ : 504
CaroS1K : ----------------MRQCCRQPQQVVMAGTA-AQAGAI-----AAAGGAVAQANQDA-AKALTHQMKRLS :  49
                           42  R    V   GT   Q   6     AA  G  A   Q      L   6 4

               *       520         *       540         *       560         *
CaroS4K : G-MIGSGKIVGKDVDILFSIPVKD-IPGYKS----PTNLDDLAKKNGNLDLPIRLAF-SDENGERVLRAFK- : 568
CaroS1K : GPSIWQGQLQMAQSFLLMGALIQHHLKGEKADLLTPEKLLEAANKQGTVPSRVRYQWMEDEETGR-LKAVGY : 120
          G  I  G 6      6L    6   6 G K     P  L   A K G 6   6R  5  DE   R L4A

           580         *       600         *       620         *       640
CaroS4K : AGSLRVP-SSVRGVAGSYDKNTGIFSAEIDGVSSRLVLENPVFPPTGNVGNTGN-TAPDYKALLNTGVDVKP : 638
CaroS1K : HTSMESGRDQVRVRLLKYDFPNNRYAFWEEGATGPTILWTPDNPGMELPTDTAHGEQPVIPSAV-PGFEIPE : 191
            S6       VR     YD     5     G 3   6L  P  P      1T     P     6  G  6

           *       660         *       680         *       700         *       720
CaroS4K : VDKITVTITPVADPVDIDDYIIWLPTASGSGVEPIYVVFNSNPYGETNSKGKYSGRSFNTDKAGGAIQNLDW : 710
CaroS1K : MDDVSILATPMPEEKDFRDYILVFPENS---FPPIYIYLSKLPVNLLDVD-LYS--NF----IGRSRQG-KY : 252
          6D 636  TP6    D  DYI6  P  S     PIY6     P    1    YS   F     G   Q   5

                   *       740         *       760         *       780         *
CaroS4K : KTANIDRAGVDKVKLHTGRFAESDANKVMIDRLERILKGTIAVTDTDKRFYTHEIRELERYRNLGIKDGVVP : 782
CaroS1K : QADHMPSAAA--VKAYLKRLY-PDLRK---DKLEKMAKEVAAII-IPTEIH-QKISETYGGRNSA--DNIEL : 314
              6  A    VK    R    D  K   D4LE46 K   A6           I E    RN     D 6

               800         *       820         *       840         *       860
CaroS4K : SNQ---GEVWNNT-HT--ATLEDYKINERTESLYTPEAIKASEEQDMRESQ--------------------- : 827
CaroS1K : DSQDLRGH-WIVILHAIKPQLKEHGVTEEQLELARDKMHKLNEEQGLY------------------------ : 361
            Q   G  W    H     L    6 E    L      K  EEQ 6
```

Supplementary Figure S5. CaroS4K is compared to caroS1K amino acid sequences.
