# Supplementary material for: A Novel Deoxyribonuclease Low-Molecular-Weight Bacteriocin, Carocin S4, from Pectobacterium carotovorum subsp. carotovorum
Source: Microorganisms. 2023 Jul 22;11(7):1854. doi: 10.3390/microorganisms11071854 (PMC10386115; doi:10.3390/microorganisms11071854)
Supplement: Supplementary file 1 [file microorganisms-11-01854-s001.zip › Supplementary Figure S6.pdf]

Supplementary Figure S6

```
   1      10        20        30        40        50        60        70        80        90        100       110       120       130
S3 MIKYRLYAPNDGDTMTVDGGGG-WDSPVNDDRKGGNDRDNGKGGSAVDYGKNPEKQTVINPYLFFAIPPAVYLIDDVWGFTFNTAAIEVGLKKVADFALKATPVAGRLVG-ILGAIMPSDIAPDSIDPVF
S4 MIKYRLYAPNDGDTMTVDGGGGGWVS--NDDRKGGNDRNNDKGGSAVDYGKNPEKQTVINPYLFFAIPPAVYLIGDVWGFTFNTAAIEVGLKKVADFALKATPVAGRLVG-TLGAIMPSDIAPDSIDPVF
S2 MIKYRLYAPNDGDTMTVDGGGG-WDSPVNDDRKGGNDRDDGKGGSAVDFSKNPEKQAIVNPYLAIALPMPVYPIFGTPGFTINTIAIETGLAKISAVISSALPVAGRLLGVTVGAMWPSSTAPSSLDSIY

   131    140       150       160       170       180       190       200       210       220       230       240       250       260
S3 NGMQQTKMAQMAAQLAAQKGVPNIGYSVTAMPADLVSSLPVSEISSLPTAPASLLAQSVINTELSRRQLTLTKPAVNAPVANIPVVKAEKTAVPGVYSVKVIADKPALQIKLDKTQPALAKNPPKVKDDV
S4 NGMQQTKMAQMAAQLAAQKGVPNIGYSVTAMPADLVSSLPVSEISSLPTAPASLLAQSVINTELSRRQLTLTKPAVNAPVANIPVVKAEKTAVPGVYSVKVIADKPALQIKLDKTQPALAKNPPKVKDDV
S2 NQAHQ----QALAQLAAQQGVLNKGYNVTAMPAGFVSSLPVSEIKSLPTAPASLLAQSVVNTELSQRQLALTQPTTNVPVANIPVVKAEKTAVPGVYSAKIIAGEPAFQIKVDSTKPALAQNPPKVKDDV

   261    270       280       290       300       310       320       330       340       350       360       370       380       390
S3 QVSSFLSSPVADTHHAFIDFGSDHEPVYVSLSKIVTAEEEKKQVEEAKRREQEWLLRHPVAAAERKVQDAINAEAEAERGVKSAELALNNLKTSPNGLTLADPVKYPAKAEWPEIYDVPEYGDYAQIKFN
S4 QVSSFLSSPVADTHHAFIDFGSDHEPVYVSLSKIVTAEEEKKQVEEAKRREQEWLLRHPITAAERKLTEIHQVLSFAQQLKESSTATISE------------------KTKTVAVYQ-EQVNTAAKNRDN
S2 QVSSFLSTPVADTHHAFIDFGSDHEPVYVSLSKIVTAEEEKKQVAEAKRREQEWLLRHPITAAERKLTEIHQVISFAQQLKESSAATISG------------------KTKTVAVYQ-EQVNTAAKNRDN

   391    400       410       420       430       440       450       460       470       480       490       500       510       520
S3 FSVLVDSVAKLNALLTQGAGALLTDEISWRDVITKYSVNNLSQDMLNDPSETMFVLGDVYKEQALEYYGYLRSELLKSKELISNAEKSLIIAIESRKKAEQDKKTADQKLKDEQDRKRNRVNVSTFGTVQ
S4 F--YNQNRGLLSAGITGGPG---------------YPIYLALWPTMNNFHQAYFRANNAL-EQESHLLNQARSDLSKAEQLL--AENNLL------------QVETERMLAEEKEIKRNRVNVSTFGTVQ
S2 F--YNQNRGLLSAGITGGPG---------------YPIYLALWQTMNNFHQAYFRANNAL-EQESHVLNQARSDLAKAEQLL--AENNLL------------QVETERTLAEEKEIKRNRVNVSTFGTVQ

   521    530       540       550       560       570       580       590       600       610       620       630       640       650
S3 TQLSTLLSAFYAATSGSTASISQSVPSGALASFSYKPQGMIGSGKIVGKDVDILFSIPVKDIPGYKSPTNFDDLAKKNGNLDLPIRLAFSNENGERVLRAFKAGSLRVPSSVRGVVGIYDKNTGIFSAEI
S4 TQLSTLLSAFYAATSGSTASISQSVPSGELASFLYKPQGMIGSGKIVGKDVDILFSIPVKDIPGYKSPTNLDDLAKKNGNLDLPIRLAFSDENGERVLRAFKAGSLRVPSSVRGVAGSYDKNTGIFSAEI
S2 TQLSTLLSAFYAATFGSTTSISQSVPSGALASFSYKSQGMIGSGKIVGKDVDILFSIPVKDIPGYKSPTNLDDLAKKNGSLDLPIRLAFSDENGERVLRAFKAGSLRVPSSVRGVAGSYDKNTGIFSAKI

   651    660       670       680       690       700       710       720       730       740       750       760       770       780
S3 DGVPSRLVLENPAFPPIGNVGNTGNTAPDYKALLNTGVDVKPVDKITVTTTPVADPVDIDDYIIWLPTASGSGVEPIYIVFNSNPYGETNSKGKYSGRSFNTDKAGGPIQSLDWKTANIDRAGVDKVKLH
S4 DGVSSRLVLENPVFPPTGNVGNTGNTAPDYKALLNTGVDVKPVDKITVTITPVADPVDIDDYIIWLPTASGSGVEPIYVVFNSNPYGETNSKGKYSGRSFNTDKAGGAIQNLDWKTANIDRAGVDKVKLH
S2 DGVSSRLVLENPAFPPTGNVGNTGNTAPDYKALLNTGIDVKPVDKITVTVTPVAEPMEFDDYIIWTPTADGSGVEPIYVVFNDPLDSDRFTRKQLDKKYLKHAKDFGIVDTRKNSETLTKFRDAIITHLE

   781    790       800       810       820       830       840       850       860       870       880883
S3 TGRFAESDANKVMIDRLERILKGT--LAVTDTDKRFYTHEIRELERYRNLGIKDGVVPSNQGEVWNNTHTATLEDYKIDERTESLYTSEAIKASEEQDMRESK
S4 TGRFAESDANKVMIDRLERILKGT--LAVTDTDKRFYTHEIRELERYRNLGIKDGVVPSNQGEVWNNTHTATLEDYKINERTESLYTPEAIKASEEQDMRESQ
S2 EKETFEKGTYLLVKDSKVFFNPKTNNVVVMDKDNKFISGWKLDVDSQQYKNYVNNGVLR
```

Supplementary Figure S6. CaroS4K is compared to the CaroS2K and CaroS3K amino acid sequences
