# Supplementary material for: A Novel Deoxyribonuclease Low-Molecular-Weight Bacteriocin, Carocin S4, from Pectobacterium carotovorum subsp. carotovorum
Source: Microorganisms. 2023 Jul 22;11(7):1854. doi: 10.3390/microorganisms11071854 (PMC10386115; doi:10.3390/microorganisms11071854)
Supplement: Supplementary file 1 [file microorganisms-11-01854-s001.zip › Supplementary Figure S7.pdf]

Supplementary Figure S7

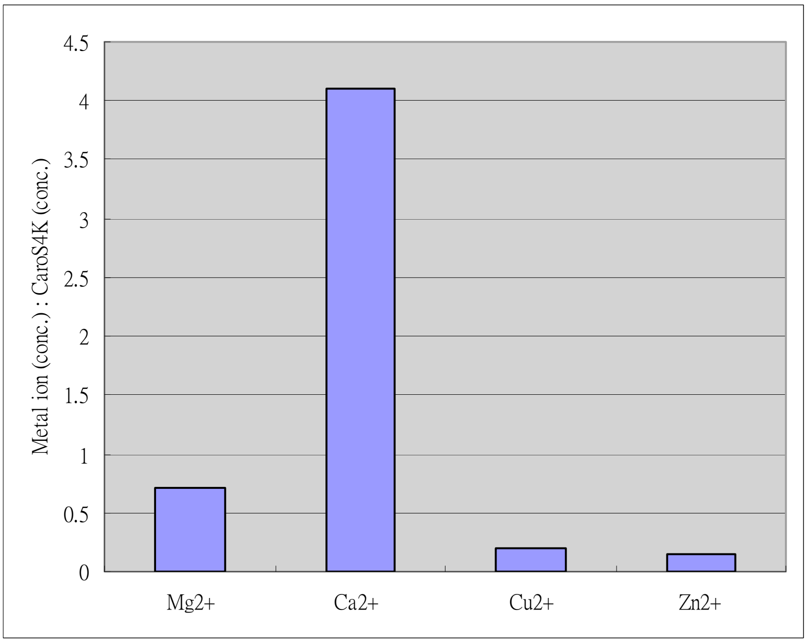

Supplementary Figure S7. CaroS4K vs. concentration ratio of major metal ions (Mg^{2+}, Ca^{2+}, Cu^{2+} and Zn^{2+}).
