# Supplementary material for: A Novel Deoxyribonuclease Low-Molecular-Weight Bacteriocin, Carocin S4, from Pectobacterium carotovorum subsp. carotovorum
Source: Microorganisms. 2023 Jul 22;11(7):1854. doi: 10.3390/microorganisms11071854 (PMC10386115; doi:10.3390/microorganisms11071854)
Supplement: Supplementary file 1 [file microorganisms-11-01854-s001.zip › Supplementary Table S1.pdf]

Supplementary Table S1. Bacteria and plasmids used in the study.

Strain or plasmid	Description	Source
Escherichia coli		
1830	*pro⁻ met⁻ Kan*^{r} *Nm*^{r}, containing transposon *Tn5* on the suicidal plasmid pBJ4JI	[19]
DH5α	supE44ΔlacU169(Φ80lacZΔM15) hsdR17recA1 gyrA96thi-1relA1	[20]
BL21(DE3)	hsdS gal(λ*c*I*ts*857 *ind*1 Sam*7 nin5 lac*UV5-T7 gene 1)	[21]
Pectobacterium carotovorum* subsp. *carotovorum		
TO-6	Pcc, wild-type	Laboratory stock
Rif-TO6	TO-6, Rif^r, wild-type	This study
TT6-3	Rif-TO6, *Carocin*S4::Tn*5*, Rif^r , Kan^r	This study
TT6-13	Rif-TO6, *Carocin*S4::Tn*5*, Rif^r , Kan^r	This study
TT6-21	Rif-TO6, *Carocin*S4::Tn*5*, Rif^r , Kan^r	This study
95F-3	Pcc, wild-type	Laboratory stock
**Plasmid**		
pMCL210	p15A, Cml^r , Low copy number	[22]
pBR322	Amp^r ; Tet^r	Promega
pGEM-T Easy	Amp^r ; lacZ, TA-cloning vector	Promega
pCRII-nptII	Amp^r ; Kan^r	Laboratory stock
pET32a	Amp^r; expression vector with the N-terminal His-tag	Novagen
pGS4KI	3032 bp DNA fragment harboring *caroS4K* and *caroS4I* genes from TO-4 genome, cloned into pGEM-T Easy Vector	This study
pGS4I	654 bp DNA fragment harboring *caroS4I* gene from TO-4 genome, cloned into pGEM-T Easy Vector	This study
pES4KI	*caroS4K* and *caroS4I* subcloned into pET32a	This study
pES4I	*caroS4I* subcloned into pET32a	This study

Kan^r: Kanamycin; Cml^r: Chloramphenicol; Rif^r: Rifampicin; Amp^r: Ampicillin.
