# Supplementary material for: A Novel Deoxyribonuclease Low-Molecular-Weight Bacteriocin, Carocin S4, from Pectobacterium carotovorum subsp. carotovorum
Source: Microorganisms. 2023 Jul 22;11(7):1854. doi: 10.3390/microorganisms11071854 (PMC10386115; doi:10.3390/microorganisms11071854)
Supplement: Supplementary file 1 [file microorganisms-11-01854-s001.zip › Supplementary Table S2.pdf]

Supplementary Table S2: Primers used in the study

Name	Sequence (5'→3')
PF-1	5′AGAGAACACAGATTTAGCCCAGTCGG 3′
PF-2	5′CCGCACGATGAAGAGCAGAAGTTAT3′
PF-3	5′GATCCTGGAAAACGGGAAAGGTTC3′
PR-1	5′GCCGAAGAGAACACAGATTTAGCCCA3′
PR-2	5′CCGCACGATGAAGAGCAGAAGTT3′
PR-3	5′CAGATCTCTGGAAAACGGGAAAGG3′
N1	5′NGTCGASWGANAWGAA3′
N2	5′GTNCGASWCANAWGTT3′
N3	5′WGTGNAGWANCANAGA3′
P3	5′CTCGACGTTGTCACTGAAGCGGGAAG3′
P4	5′AAAGCACGAGGAAGCGGTCAGCCCAT3′
TO6K-sens13	5′CAGATGATGCAGAGCATTCAGG3′
TO6I-anti7	5′GGCCGATGCTGGTTTTGTGGT3′
TO6-sens3	5′GGTGATGATTGACCGTCTTGAGAG3′
Carocin S4KI_for	5′GGATCCATGATTAAATACCG 3′
Carocin S4KI_rev	5′GAGCTCTTAGAGACCGTAT 3′
5IHT32a2KI_forT	5′GAAGGAGATATACATATGATTAAGTACCGTTTATA 3′
5IHTGT2KI_forS	5′ATGATTAAGTACCGTTTATA 3′
5IHT32a3KI_revT	5′ ATGTATATCTCCTTCTTAAAGTTAAACAAAATTATTTC 3′
5IHT32a4KI_revS	5′TTAAAGTTAAACAAAATTATTTC 3′
X4I_forT	5′GAAGGAGATATACATATGATTAATTTTAAGGCAATG 3′
X4I_forS	5′ATGATTAATTTTAAGGCAATG 3′
Carocin S4KI_for	5′GGATCCATGATTAAATACCG 3′
Carocin S4KI_rev	5′GAGCTCTTAGAGACCGTAT 3′
5IHT32a2KI_forT	5′GAAGGAGATATACATATGATTAAGTACCGTTTATA 3′
5IHTGT2KI_forS	5′ATGATTAAGTACCGTTTATA 3′
